# Alcohol dependence trajectories and smoking cessation among Korean men who smoke: A secondary data analysis from the Korean longitudinal study of aging dataset

**DOI:** 10.18332/tid/205795

**Published:** 2025-07-23

**Authors:** Minjung Han, Heewon Kang, Hae-ryoung Chun, Sung-il Cho

**Affiliations:** 1 Department of Public Health Sciences, Graduate School of Public Health, Seoul National University, Seoul, Republic of Korea; 2 Department of Family Medicine, Korea University Ansan Hospital, Korea University College of Medicine, Ansan, Republic of Korea; 3 Institute of Health and Environment, Graduate School of Public Health, Seoul National University, Seoul, Republic of Korea; 4 Department of Psychiatry, College of Medicine, The Catholic University of Korea, Seoul, Republic of Korea

**Keywords:** alcohol dependence, smoking cessation, longitudinal study, Korean men, latent class growth analysis

## Abstract

**INTRODUCTION:**

Alcohol dependence may hinder smoking cessation, yet few studies have examined how long-term patterns of alcohol use influence quit outcomes. This study assessed how alcohol dependence trajectories affect smoking cessation among Korean men who smoke.

**METHODS:**

We performed a secondary analysis using waves 1–7 (2006–2018) of the Korean Longitudinal Study of Aging (KLoSA). Latent class growth analysis (LCGA) identified alcohol dependence trajectories among 2356 men aged ≥45 years who participated in at least three consecutive waves. Multinomial logistic regression (n=1959) was used to assess predictors of trajectory class membership, and Cox proportional hazards models (n=1122) were used to evaluate the association between class membership and smoking cessation. Statistical significance was set at a two-sided p<0.05.

**RESULTS:**

Three alcohol dependence trajectories were identified: stable low (80.7%), decreasing (14.3%), and increasing (5.0%). Participants in the decreasing (adjusted hazard ratio, AHR=0.77; 95% CI: 0.63–0.95) and increasing (AHR=0.60; 95% CI: 0.42–0.86) groups were less likely to quit smoking than the stable low group. Multinomial regression showed that, compared to non-smokers, both former smokers (AOR=1.83; 95% CI: 1.24–2.70) and current smokers (AOR=2.23; 95% CI: 1.60–3.09) were associated with higher odds of belonging to the decreasing trajectory. Only current smoking was significantly associated with the increasing trajectory (AOR=2.28; 95% CI: 1.36–3.84). In stratified analyses, the inverse association between increasing trajectory and quitting was significant only in those aged 45–54 years. Sensitivity analyses using weighted and complete-case data confirmed the robustness of the findings.

**CONCLUSIONS:**

Alcohol dependence trajectories were significantly associated with smoking cessation outcomes, especially among younger individuals. Smoking status was also a significant predictor of trajectory class membership, with current smokers more likely to belong to the increasing trajectory. Integrated interventions addressing both behaviors may improve cessation outcomes in high-risk groups.

## INTRODUCTION

Harmful alcohol use remains a significant global public health concern. In 2016, alcohol consumption contributed to approximately 3 million deaths worldwide (5.3% of all deaths) and accounted for 132.6 million disability-adjusted life years (DALYs), or 5.1% of the global disease burden^1^. Alcohol use disproportionately affects men, accounting for an estimated 2.3 million deaths and 106.5 million DALYs, compared to 0.7 million deaths and 26.1 million DALYs among women^1^. Globally, around 237 million men and 46 million women suffer from alcohol use disorders (AUD), which encompasses alcohol dependence, abuse, and addiction^2^.

Individuals with AUD show considerable heterogeneity^3^. Using National Epidemiological Survey on Alcohol and Related Conditions (NESARC) data, Moss et al.^4^ identified five subtypes of alcohol dependence based on cross-sectional data that were characterized by differences in family history, age of onset, comorbidities, and AUD criteria. Moreover, longitudinal studies have demonstrated that alcohol use patterns can vary significantly over time across individuals with different characteristics^5,6^. For instance, in a prospective cohort study of US adults, the rate of change in heavy drinking became slower for those who entered into marriage or quit smoking during the study period^5^. Moreover, a latent growth mixture modeling approach using longitudinal data identified alcohol use trajectories from young adulthood to midlife^6^. This analysis revealed substantial heterogeneity in alcohol use patterns, which were associated with factors such as early onset, solitary drinking, and psychiatric morbidity^6^.

Alcohol dependence is often accompanied by other substance use, most notably smoking^7,8^. There is consistent evidence that alcohol dependence can hinder smoking cessation by reducing quit success and increasing relapse risk^9^. For instance, a prospective longitudinal study of US adults aged 55–65 years found that episodic heavy drinking at baseline was associated with an increased percentage of follow-ups with continued smoking and with current smoking at the participants’ last observation^10^. Another study of US adults aged ≥50 years from the 1992–2012 Health and Retirement Study reported that heavy drinkers who did not smoke at baseline were 60% more likely to start smoking during the follow-up period, compared to those without heavy drinking (heavy drinking defined as ≥1 drink/day or ≥4/occasion for women; ≥2 drinks/day or ≥4/occasion for men, past 3 months)^11^. Additionally, longitudinal research reported bidirectional associations between alcohol use disorder (AUD) and tobacco dependence (TD), with each predicting the other at various follow-up points^12^. These findings demonstrate the close relationship between alcohol dependence and smoking behaviors, and indicate the importance of addressing both in cessation efforts.

Despite evidence for the heterogeneity of usage patterns over time among individuals with alcohol dependence and the close relationship between alcohol dependence and quit behavior, few studies have examined how smoking cessation outcomes differ across subgroups characterized by distinct alcohol dependence trajectories. Given the paucity of such studies, we focused on investigating smoking cessation outcomes according to alcohol trajectory groups in Korean men who smoke. The analysis was restricted to men only, due to the low prevalence rates among Korean women^13^. By identifying different alcohol dependence trajectory classes and how these groups differ in smoking cessation outcomes, we may be able to provide evidence for integrated treatment for alcohol use disorders in high-risk populations, especially those that display high dependence on both alcohol and nicotine and have consistently shown low quit success rates.

This study aims to: 1) identify distinct trajectories of alcohol dependence over time among Korean adult men using KLoSA data; 2) describe the baseline characteristics associated with each trajectory; and 3) examine the association between alcohol dependence trajectory class and smoking cessation outcomes.

## METHODS

### Data and sample

Data were obtained from the 1st to the 7th wave (2006–2018) of the Korean Longitudinal Study of Aging (KLoSA)^14^. KLoSA is a nationally representative panel survey of community dwelling adults aged ≥45 years in South Korea. KLoSA has been conducted every two years by the Korea Labor Institute of the Ministry of Labor since 2006. Its purpose is to support policymaking and institutional research regarding the aging process in Koreans.

The survey employed a stratified, multistage sampling design, with the selection of samples based on probability proportional to size and geographical areas. Interviews were conducted face-to-face with the aid of computer-assisted personal interviewing method. KLoSA includes details on sociodemographics, health status, family support, social engagement and life satisfaction. The data are publicly available on its website^15^. Details of the study design are described elsewhere^16^.

**Figure 1:**
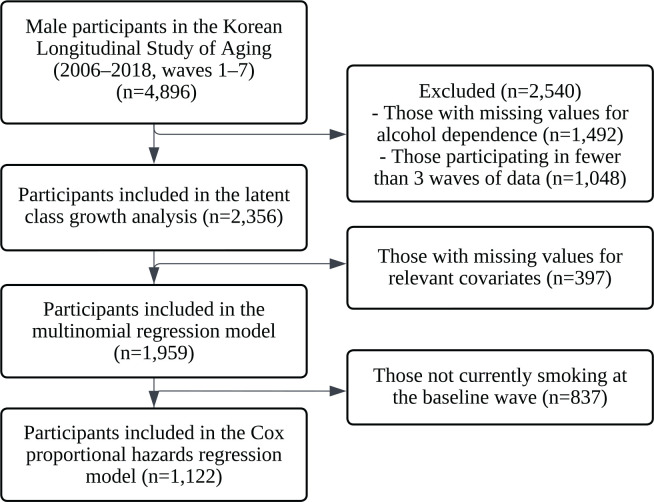
Selection of study participants

This is a longitudinal observational study based on secondary data analysis of KLoSA. It aimed to examine the relationship between alcohol dependence trajectories and smoking cessation among men aged ≥45 years who currently smoked at the baseline wave. For the latent class growth analysis (LCGA), we included men aged ≥45 years at the baseline wave who had no missing data on alcohol dependence and smoking status, and had participated in at least three consecutive waves. A total of 2356 participants met these criteria and were included in the LCGA (Figure 1). For the multinomial logistic regression analysis, which required complete data on the covariates used for adjustment, 1959 participants were included (Figure 1). For the Cox proportional hazards regression model that examined the association between alcohol dependence trajectories and smoking cessation, the sample was further restricted to 1122 participants who, in addition to the previous criteria, currently smoked at baseline, had complete data for all covariates of interest, and participated in at least three consecutive waves (Figure 1). The 1122 participants included in the Cox proportional hazards regression model were drawn from those in the multinomial regression analysis, which in turn comprised a subset of the LCGA sample (Figure 1). This study was exempt from approval by the Institutional Review Board (IRB) at Seoul National University (IRB No. E2504/003-015).

### Imputation of missing data

We used imputed values computed by the KLoSA research group using multiple imputation methods^14^. For continuous and count-type variables, five-fold multiple imputation was performed using sequential regression methods (IVEware; University of Michigan). For categorical variables, only single imputation was performed to avoid implausible category changes across imputations. Details of the imputation and variables involved are documented in the official KLoSA Multiple Imputation Manual^14^. Since our main exposure, alcohol dependence, was derived from a categorical variable, we performed LCGA using only the first imputed dataset. As a sensitivity check, we repeated LCGA across all imputed datasets to evaluate the consistency of model-derived class membership, class proportions, and model fit statistics.

### Study variables

Drinking behavior was categorized into three groups: normal, heavy drinking and alcohol dependence. These groups were classified based on responses to the CAGE questionnaire, which is an assessment widely used to identify alcohol dependence^17^. The questionnaire includes the following questions: ‘Have you ever felt that you need to cut down on your drinking?’, ‘Have you ever been annoyed by others criticizing your drinking?’, ‘Have you ever felt bad or guilty about your drinking?’, and ‘Have you ever had a drink first thing in the morning to steady your nerves or to get rid of a hangover (eye opener)?’^18^. The respondent who answered yes to one of the CAGE questions was considered as having heavy drinking; the respondent who answered yes to at least two of the CAGE questions was considered to have alcohol dependence^19^.

Smoking status was defined based on self-report of smoking history provided by the participants. Those who answered yes to the question, ‘Are you currently smoking’ were classified as current smokers. Those not currently smoking but who have smoked more than 5 packs (100 cigarettes) in their lifetime were classified as former smokers. Those who have not smoked at least 5 packs (100 cigarettes) in their lifetime and who responded that they were not currently smoking were classified as non-smokers.

In terms of defining smoking cessation, a participant was considered to have quit smoking if they reported a status of former smoker at any later wave compared to the baseline wave, at which all participants were current smokers. If the participant never reported a smoking status of formerly smoking during follow-up, they were censored at their last available wave. Follow-up time was defined as the time difference in years between the minimum wave when former smoker status was reported and the baseline wave. If the participant never quit, the follow-up time was the difference between the final wave of participation and the baseline wave.

Other covariates included sociodemographic and health-related variables. Age was treated as a continuous and categorical variable (≥45 to <55, ≥55 to <65, ≥65 years). Education level was classified into four levels (elementary school or lower, middle school graduate or lower than high school, high school graduate or lower than college, college graduate or higher). Marital status was classified into two levels: married and unmarried (including widowed, separated, divorced and never married). Employment status was classified into two levels: employed and unemployed. Monthly household income (in thousand KRW) was classified into four quartile-based groups as follows: 1st quartile (lowest income group) <300; 2nd quartile between 300 and <1200; 3rd quartile between 1200 and <3000; and the 4th quartile (highest income group) ≥3000. Depressive symptoms were measured by a 10-item version of the Center for Epidemiological Studies Depression Scale^20^. Participants were classified as having depressive symptoms if their total score exceeded 20, resulting in a binary classification of depression status. Self-rated health was defined as the response to the question, ‘How do you rate your health status?’. The possible responses were ‘very good’, ‘good’, ‘fair’, ‘bad’, and ‘very bad’. This five-category scale was transformed into three groups by changing very good and good to ‘good’, fair to ‘fair’ and bad and very bad to ‘poor’.

### Statistical analysis

**Figure 2:**
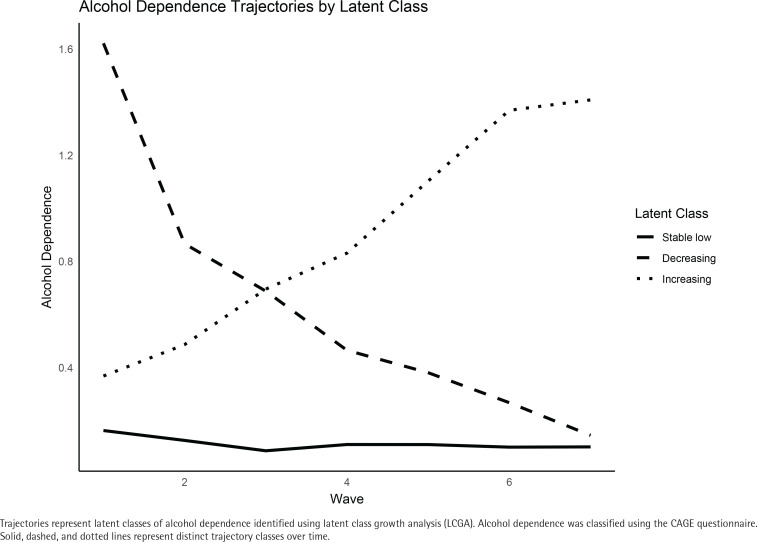
Alcohol dependence trajectories identified by latent class growth analysis among Korean men aged ≥45 years, KLoSA (N=2356)

Latent Class Growth Analysis (LCGA) was conducted to identify distinct trajectories of alcohol dependence over time. LCGA is a person-centered approach that groups individuals into mutually exclusive classes based on similarities in their longitudinal response patterns^21^. In this study, the LCGA model was fit based on alcohol dependence assessed longitudinally across consecutive survey waves (Figure 2). We determined the optimal number of alcohol dependence trajectory groups by comparing fit indices across models with increasing numbers of classes. The criteria included the Akaike Information Criterion (AIC) and Bayesian Information Criterion (BIC), with lower values suggesting better model fit. Additionally, we compared the log-likelihood values across models, as higher log-likelihood values indicate improved model fit. We also considered entropy as an indicator of classification precision; values ≥0.80 were taken to indicate good separation between classes. To ensure the robustness of the selected solution, we examined the approximate Lo-Mendell-Rubin likelihood ratio test (LMRT), which tests whether the model with one additional class significantly improves fit compared to the previous model. Finally, to assess the certainty of the classifications, we examined the average posterior probabilities within each assigned class.

Following the identification of alcohol dependence trajectory classes, we performed a multinomial logistic regression to examine the baseline variables associated with membership in each class. Adjusted odds ratios (AORs) with 95% confidence intervals (CI) were calculated to assess the association between each variable and class membership. For each identified class, we first calculated the quit rates for smoking cessation over the follow-up period and tested for significant differences between classes using a chi-squared test. We then conducted a Kaplan–Meier survival analysis with a log-rank test to compare the probability of smoking cessation between classes and generated survival curves. Subsequently, we used class membership derived from latent class growth analysis (LCGA) in a Cox proportional hazards model to calculate the unadjusted and adjusted hazard ratios (AHRs) and 95% confidence intervals (CIs) for the association between alcohol dependence trajectories and smoking cessation. The multivariable model was adjusted for age, education level, income, marital status, employment status, depression, and self-rated health, which were selected based on their established associations with both alcohol dependence and smoking cessation in previous studies. We tested the proportional hazards assumption using Schoenfeld residual tests, which indicated that the assumption was satisfied for all models. All analyses used unweighted data, following previous KLoSA-based studies that examined longitudinal trajectories or identified latent classes^22,23^. All statistical analyses were performed using R software (version 4.4.2; R Foundation for Statistical Computing, Vienna, Austria). A two-tailed p<0.05 was considered statistically significant.

### Sensitivity analyses

To assess the robustness of our findings, we conducted two sets of sensitivity analyses. First, we repeated the main analyses (multinomial logistic regression and Cox proportional hazards models) using weighted data to account for the complex sampling design.

Subsequently, we conducted complete-case analyses using non-imputed data, restricting the sample to participants without missing data on covariates used in the adjusted models. This allowed us to assess the potential influence of multiple imputation on our results. Results from both the weighted and complete-case analyses were compared against the original (unweighted, imputed) results.

### Stratified analysis by age group

To assess whether the association between alcohol dependence trajectory class and smoking cessation varied by age, we conducted a stratified analysis by age group (45–54, 55–64, and ≥65 years). We ran separate Cox proportional hazards models within each stratum that were adjusted for the same covariates as the main model, including education level, income, marital status, employment status, depression, and self-rated health.

## RESULTS

Table 1 shows the baseline characteristics of the study participants. At baseline, the mean age of the study participants was 58.3 years (SD=9.3). Of the study participants: 38.1% had an education level of a high school graduate or lower than a college degree; 93.6% were married; 70.2% were employed; 30.0% had an income in the third quartile (between 1200 and <3000 thousand KRW); 5.9% had depression; 54.6% reported good health status; 30.5% were non-smokers, 20.5% were former smokers, and 49.0% current smokers; and 71.6% had normal drinking behavior, 16.8% were heavy drinkers and 11.6% had alcohol dependence.

**Table 1:** Baseline characteristics of Korean men aged ≥45 years included in the multinomial regression analysis in the KLoSA (N=1959)

*Characteristics*	*Category*	*n (%)*
**Age** (years), mean ± SD		58.3 ± 9.3
**Age** (years)	≥45 to <55	799 (40.8)
	≥55 to <65	626 (32.0)
	≥65	534 (27.3)
**Education level**	Elementary school or lower	524 (26.7)
	Middle school graduate or lower than high school	341 (17.4)
	High school graduate or lower than college	746 (38.1)
	College graduate or higher	348 (17.8)
**Marital status**	Married	1834 (93.6)
	Unmarried	125 (6.4)
**Employment status**	Employed	1376 (70.2)
	Unemployed	583 (29.8)
**Income group**	First quartile (lowest)	373 (19.0)
	Second quartile	416 (21.2)
	Third quartile	588 (30.0)
	Fourth quartile (highest)	582 (29.7)
**Depression**	No	1844 (94.1)
	Yes	115 (5.9)
**Self-reported health**	Good	1069 (54.6)
	Fair	617 (31.5)
	Poor	273 (13.9)
**Smoking status**	Non-smoker	597 (30.5)
	Former smoker	402 (20.5)
	Current smoker	960 (49.0)
**Alcohol dependence**	Normal	1402 (71.6)
	Heavy drinking	330 (16.8)
	Alcohol dependence	227 (11.6)

Frequencies are unweighted. Monthly income (in KRW) is classified into quartiles: 1st (<300K), 2nd (300 to <1200K), 3rd (1200 to <3000K), and 4th (≥3000K). KRW: 1000 Korean Won about US$0.74.

LCGA was performed to determine the alcohol dependence trajectories of the study participants. Table 2 presents the AIC, BIC, log-likelihood, entropy and the proportions of participants in each latent class. The 3-class model demonstrated the lowest BIC and the highest log-likelihood, which indicated superior model fit compared to the 1-class and 2-class models (Table 2). Also, the 3-class model had an entropy value of 0.83, which demonstrated good classification quality (Table 2). The Lo-Mendell-Rubin likelihood ratio test (LMRT) comparing the 2-class and 3-class solutions indicated a significantly better fit for the 3-class model (χ^2^=501.91, df=4, p<0.001). To further assess the quality of classification, we examined the average posterior probabilities for each assigned class. The mean probabilities were 94.9% for class 1, 86.9% for class 2, and 84.6% for class 3 (Supplementary file Table S2), which showed strong certainty in class assignments. Each trajectory group was labeled as follows: stable low (class 1), decreasing (class 2), and increasing (class 3). The proportions of participants within each class were 80.7% (n=1903) in class 1, 14.3% (n=336) in class 2 and 5.0% (n=117) in class 3 (Table 2).

**Table 2:** Model fit statistics for latent class analysis of alcohol dependence trajectories among Korean men aged ≥45 years, KLoSA (N=2356)

*Model*	*Log likelihood*	*AIC*	*BIC*	*Entropy*	*Class 1* *n (%)*	*Class 2* *n (%)*	*Class 3* *n (%)*
1-class	-9846.8	19701.6	19724.6	1	2356 (100)	-	-
2-class	-9148.6	18313.2	18359.3	0.77	1973 (83.7)	383 (16.3)	-
3-class	-8897.6	17819.2	17888.4	0.83	1903 (80.7)	336 (14.3)	117 (5.0)

AIC: Akaike Information Criterion. BIC: Bayesian Information Criterion. Entropy measures classification certainty (values closer to 1 indicate clearer classification).

Chi-squared tests across the alcohol dependence trajectory classes indicated that there were significant differences in the following demographic and socioeconomic factors: education (p<0.001), marital status (p=0.023), income (p<0.01), depression (p<0.001), self-rated health (p<0.001) and smoking status (p<0.001) (Table 3). In terms of smoking status, there was a significantly higher proportion of those currently smoking in class 3, the class with increasing alcohol dependence over time (Table 3).

**Table 3:** Comparison of variables by alcohol dependence trajectory class among Korean men aged ≥45 years, using chi-squared tests, KLoSA (N=1959)

*Variable*	*Class 1 (Stable low)* *(N=1551)* *n (%)*	*Class 2 (Decreasing)* *(N=307)* *n (%)*	*Class 3 (Increasing)* *(N=101)* *n (%)*	*p*
**Age** (years), mean ± SD	58.4 ± 9.5	58.4 ± 8.9	57.0 ± 8.5	0.32
**Age** (years)				0.52
≥45 to <55	634 (79.3)	118 (14.8)	47 (5.9)	
≥55 to <65	488 (78.0)	106 (17.0)	32 (5.1)	
≥65	429 (80.3)	83 (15.5)	22 (4.2)	
**Education level**				<0.001
Elementary school or lower	388 (74.0)	109 (20.8)	27 (5.2)	
Middle school graduate or lower than high school	257 (75.4)	65 (19.1)	19 (5.6)	
High school graduate or lower than college	616 (82.6)	89 (11.9)	41 (5.5)	
College graduate or higher	290 (83.3)	44 (12.6)	14 (4.0)	
**Marital status**				0.023
Married	1457 (79.4)	289 (15.8)	88 (4.8)	
Unmarried	94 (75.2)	18 (14.4)	13 (10.4)	
**Employment status**				0.63
Employed	1097 (79.7)	211 (15.3)	68 (4.9)	
Unemployed	454 (77.9)	96 (16.5)	33 (5.7)	
**Income group**				<0.01
First quartile (lowest)	307 (82.3)	50 (13.4)	16 (4.3)	
Second quartile	315 (75.7)	77 (18.5)	24 (5.8)	
Third quartile	439 (74.7)	113 (19.2)	36 (6.1)	
Fourth quartile (highest)	490 (84.2)	67 (11.5)	25 (4.3)	
**Depression**				<0.001
No	1479 (80.2)	275 (14.9)	90 (4.9)	
Yes	72 (62.6)	32 (27.8)	11 (9.6)	
**Self-rated health**				<0.001
Good	882 (82.5)	133 (12.4)	54 (5.0)	
Fair	482 (78.1)	107 (17.3)	28 (4.5)	
Poor	187 (68.5)	67 (24.5)	19 (6.9)	
**Smoking status**				<0.001
Non-smoker	522 (87.4)	55 (9.2)	20 (3.3)	
Former smoker	323 (80.3)	67 (16.7)	12 (3.0)	
Current smoker	706 (73.5)	185 (19.3)	69 (7.2)	

Frequencies are unweighted. Monthly income (in KRW) is classified into quartiles: 1st (<300K), 2nd (300 to <1200K), 3rd (1200 to <3000K), and 4th (≥3000K). KRW: 1000 Korean Won about US$0.74. Statistical significance for the chi-squared test was assessed using a two-sided p-value threshold of <0.05.

Table 4 presents the class-specific odds ratios from the multinomial regression, conducted to identify the factors associated with membership in each alcohol dependence trajectory class. Compared to non-smokers, former smokers (AOR=1.83; 95% CI: 1.24–2.70) and current smokers (AOR=2.23; 95% CI: 1.60–3.09) were associated with significantly higher odds of being in class 2. In contrast, compared to non-smoking, only current smoking was significantly associated with higher odds of class 3 membership (AOR=2.28; 95% CI: 1.36–3.84), while former smoking was not (AOR=0.94; 95% CI: 0.45–1.97).

**Table 4:** Multinomial logistic regression predicting alcohol dependence trajectory class membership among Korean men aged ≥45 years, KLoSA (N=1959)

*Variable*	*Class 2 (Decreasing)* *(ref: Stable low)*			*Class 3 (Increasing)* *(ref: Stable low)*		
	*AOR*	*95% CI*	*p*	*AOR*	*95% CI*	*p*
**Age** (years)						
≥45 to <55 ®	1			1		
≥55 to <65	0.93	0.68–1.28	0.67	0.84	0.51–1.40	0.50
≥65	0.69	0.47–1.03	0.07	0.62	0.32–1.18	0.14
**Education level**						
Elementary school or lower ®	1			1		
Middle school graduate or lower than high school	0.85	0.59–1.22	0.38	0.99	0.52–1.87	0.98
High school graduate or lower than college	0.57	0.40–0.80	**<0.001**	1.02	0.58–1.81	0.94
College graduate or higher	0.69	0.45–1.06	0.09	0.82	0.39–1.71	0.59
**Marital status**						
Married ®	1			1		
Unmarried	0.69	0.4–1.19	0.18	1.72	0.88–3.33	0.11
**Employment status**						
Employed ®	1			1		
Unemployed	0.99	0.73–1.35	0.96	1.27	0.77–2.10	0.35
**Income group**						
First quartile (lowest)	1			1		
Second quartile	1.43	0.96–2.14	0.08	1.48	0.76–2.88	0.25
Third quartile	1.75	1.19–2.58	**<0.001**	1.70	0.90–3.21	0.10
Fourth quartile (highest)	1.08	0.7–1.67	0.73	1.18	0.58–2.41	0.64
**Depression**						
No ®	1			1		
Yes	1.86	1.15–3.03	**0.01**	1.92	0.91–4.09	0.09
**Self-rated health**						
Good ®	1			1		
Fair	1.38	1.03–1.84	**0.03**	0.96	0.59–1.56	0.86
Poor	1.96	1.34–2.87	**<0.001**	1.43	0.76–2.70	0.27
**Smoking status**						
Non-smoker ®	1			1		
Former smoker	1.83	1.24–2.70	**<0.001**	0.94	0.45–1.97	0.88
Current smoker	2.23	1.60–3.09	**<0.001**	2.28	1.36–3.84	**<0.001**

AOR: adjusted odds ratio; adjusted for age, education level, marital and employment status, income, depression, self-rated health, and smoking status. Monthly income (in KRW) is classified into quartiles: 1st (<300K), 2nd (300 to <1200K), 3rd (1200 to <3000K), and 4th (≥3000K). KRW: 1000 Korean Won about US$0.74. Statistical significance for the chi-squared test was assessed using a two-sided p-value threshold of <0.05. ® Reference categories.

Several other factors were significantly associated with class 2 (decreasing) membership. Compared to those with an elementary school education or lower, high school graduates were less likely to belong to class 2 (AOR=0.57; 95% CI: 0.40–0.80). Participants in the third quartile of income had higher odds of being in class 2 than those in the first quartile (AOR=1.75; 95% CI: 1.19–2.58). Those with depression had higher odds of being in class 2 (AOR=1.86; 95% CI: 1.15–3.03). Compared to those with good self-rated health, those with fair self-rated health had increased odds of being in class 2 (AOR=1.38; 95% CI: 1.03–1.84), while those with poor health had even greater odds of being in class 2 (AOR=1.96; 95% CI: 1.34–2.87).

In addition, we examined the smoking quit rates in each alcohol dependence trajectory class. Descriptive quit rates demonstrated that class 1 (stable low) had the highest quit rate for smoking (66.7%), followed by class 2 (decreasing), which had a quit rate of 58.4% (Table 5). Class 3 (increasing) had the lowest quit rate at 44.6%. This difference was statistically significant (χ^2^=17.0, p <0.001).

**Table 5:** Smoking cessation rates by alcohol dependence trajectory class among Korean men aged ≥45 years who currently smoked at baseline, KLoSA (N=1122)

*Alcohol dependence trajectory class*	*Total* *n*	*Quitters* *n*	*Quit rate* *%*
Class 1 (Stable low)	846	564	66.7
Class 2 (Decreasing)	202	118	58.4
Class 3 (Increasing)	74	33	44.6
Chi-squared test			χ²=17.0, df=2, p=0.0002

Statistical significance was assessed using a two-sided p-value threshold of <0.05.

**Figure 3:**
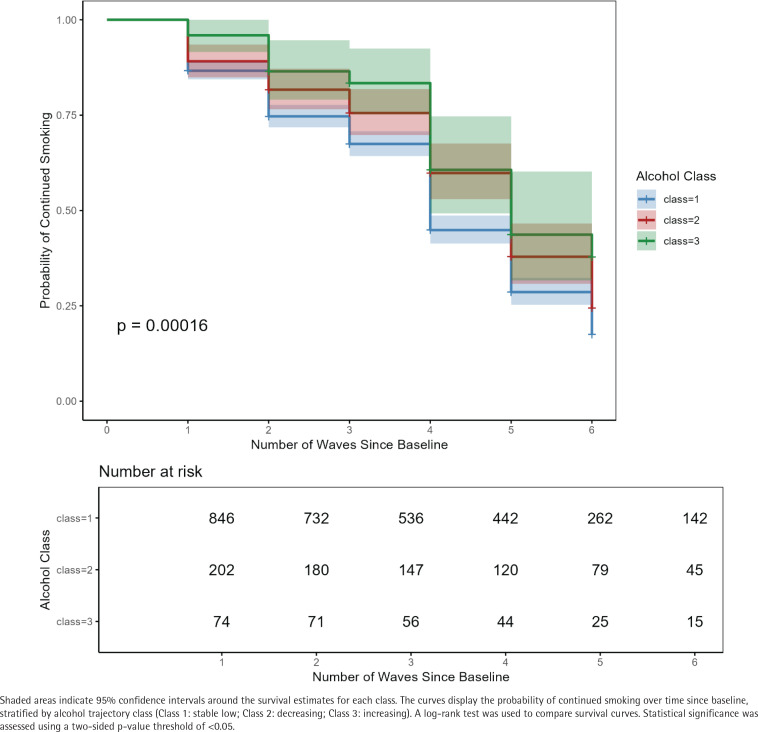
Kaplan–Meier curves of time to smoking cessation by alcohol dependence trajectory class, for Korean men aged ≥45 years who currently smoked at baseline, KLoSA (N=1122)

Subsequently, we used a Cox proportional hazards model to evaluate the association between alcohol dependence trajectories and smoking cessation. Over the study period, with a mean follow-up of 7.2 years (SD=3.4), 715 out of 1122 individuals quit smoking, contributing a total of 7986 person-years (PYs) of follow-up. The incidence rates for classes 1 through 3 were 9.5, 7.7 and 5.8 per 100 person-years, respectively, with significant differences observed between groups (log-rank test, p<0.001) (Figure 3). In both unadjusted and fully adjusted Cox proportional hazards models, alcohol dependence trajectory was a significant determinant of smoking cessation (Table 6). In the fully adjusted Cox proportional hazards model, compared to class 1, those in class 2 had a 23% lower likelihood of quitting smoking (AHR=0.77; 95% CI: 0.63–0.95). Also, participants in class 3 had a 40% lower likelihood of quitting smoking compared to class 1 (AHR=0.60; 95% CI: 0.42–0.86). Older age (55–64 and ≥65 years) was associated with higher likelihood of quitting (55–64 years: AHR =1.34; 95% CI: 1.11–1.60; and ≥65 years: AHR=1.48; 95% CI: 1.16–1.90). Higher income (fourth quartile) was associated with higher likelihood of quitting (AHR=1.47; 95% CI: 1.15–1.87). Depression, self-rated health, employment, and education were not significantly associated with quitting after adjustment.

**Table 6:** Cox proportional hazards model predicting time to smoking cessation by alcohol dependence trajectory class among Korean men aged ≥45 years who currently smoked at baseline, KLoSA (N=1122)

*Variable*	*Unadjusted*			*Adjusted*		
	*HR*	*95% CI*	*p*	*AHR*	*95% CI*	*p*
**Class** (ref: Stable low)						
Class 2 (Decreasing)	0.75	0.62–0.92	**<0.01**	0.77	0.63–0.95	**0.013**
Class 3 (Increasing)	0.57	0.40–0.81	**<0.01**	0.60	0.42–0.86	**<0.01**
**Age** (years) (ref: 45–54)						
55–64				1.34	1.11–1.60	**<0.01**
≥65				1.48	1.16–1.90	**<0.01**
**Education level** (ref: Elementary school or lower)						
Middle school graduate or lower than high school				0.99	0.78–1.25	0.93
High school graduate or lower than college				1.04	0.85–1.29	0.69
College graduate or higher				1.15	0.89–1.49	0.27
**Marital status** (ref: Married)						
Unmarried				0.85	0.63–1.14	0.28
**Income** (ref: First quartile, lowest)						
Second quartile				0.98	0.77–1.25	0.88
Third quartile				1.08	0.85–1.37	0.51
Fourth quartile				1.47	1.15–1.87	**<0.01**
**Employment** (ref: Employed)						
Unemployed				1.06	0.88–1.29	0.53
**Depression** (ref: No depression)						
Depression				1.28	0.94–1.76	0.12
**Self-rated health** (ref: Good)						
Fair				0.90	0.76–1.06	0.20
Poor				0.88	0.68–1.14	0.33

AHR: adjusted hazard ratio; adjusted for alcohol dependence trajectory class, age, income, marital and employment status, depressive symptoms, and self-rated health. Income (in KRW) is classified into quartiles: 1st (<300K), 2nd (300 to <1200K), 3rd (1200 to <3000K), and 4th (≥3000K). KRW: 1000 Korean Won about US$0.74. Statistical significance for the chi-squared test was assessed using a two-sided p-value threshold of <0.05.

### Sensitivity analyses

Sensitivity analyses across the multiple imputation sets showed that class proportions and model fit statistics remained consistent (Supplementary file Table S1). These results suggest that the first imputed dataset provides a representative and stable basis for conducting LCGA and assigning class membership.

Furthermore, results from the Cox proportional hazards model with weighted and complete-case data were largely consistent with the findings from the original analysis. The association between alcohol dependence trajectory classes and smoking cessation was robust in the weighted models (class 2, AHR=0.74; 95% CI: 0.64–0.84; and class 3, AHR=0.80; 95% CI: 0.72–0.88), but not significant in the complete-case models (class 2, AHR=0.87; 95% CI: 0.68–1.10; and class 3, AHR=0.70; 95% CI: 0.47–1.06) (Table 6; and Supplementary file Tables S4 and S6). Also, age was consistently significant and associated with higher likelihood of cessation in the weighted models (55–64 years, AHR=1.45; 95% CI: 1.32–1.59; and ≥65 years, AHR=1.51; 95% CI: 1.33–1.72) as well as in the complete-case models (55–64 years, AHR=1.35; 95% CI: 1.09–1.67; and ≥65 years, AHR=1.62; 95% CI: 1.21–2.16).

Also, in the multinomial regression model, sensitivity analyses using both weighted data and complete-case data confirmed the robustness of the original results (Table 4; and Supplementary file Tables S3 and S5). In all models, including the original, weighted, complete-case models, compared to non-smoking, current smoking significantly increased the likelihood of belonging to class 3 (original AOR=2.28; 95% CI: 1.36–3.84; weighted AOR=1.84; 95% CI: 1.59–2.15; complete-case AOR=2.27; 95% CI: 1.35–3.83) (Table 4; and Supplementary file Tables S3 and S5). Compared to non-smoking, former smoking was associated with increased likelihood of class 3 membership only in the weighted model (AOR=1.63; 95% CI: 1.36–1.96) (Supplementary file Table S3). Smoking status was also a significant predictor of membership in class 2 across all models, with both former smoking (original, AOR=1.83; 95% CI: 1.24–2.70; weighted, AOR=1.78; 95% CI: 1.34–2.35; complete-case, AOR=1.83; 95% CI: 1.24–2.71) and current smoking (original, AOR=2.23; 95% CI: 1.60–3.09; weighted AOR=2.04; 95% CI: 1.65–2.52; complete-case, AOR=2.25; 95% CI: 1.62–3.12) associated with an increased likelihood of membership in class 2 (Table 4; and Supplementary file Tables S3 and S5).

### Stratified analysis of the association between alcohol dependence trajectory class and smoking cessation by age group

When the Cox proportional hazards model was stratified by age group, we found a differential association between alcohol dependence trajectory class and cessation by age group (Supplementary file Table S7). In those aged 45–54 years, class 2 (AHR=0.69; 95% CI: 0.50–0.96) and class 3 (AHR=0.47; 95% CI: 0.28–0.79) were both significantly associated with lower likelihood of cessation. In those aged 55–64 years, class 2 remained significant for lower likelihood of cessation (AHR=0.67; 95% CI: 0.47–0.96), but class 3 was not (AHR=0.64; 95% CI: 0.31–1.32). Lastly, in those aged ≥65 years, neither class 2 (AHR=1.17; 95% CI: 0.77–1.80) nor class 3 (AHR=1.10; 95% CI: 0.54–2.23) were significantly associated with smoking cessation.

## DISCUSSION

This study used longitudinal data from KLoSA to identify three distinct alcohol dependence trajectories among Korean men aged ≥45 years based on CAGE criteria: stable low (class 1), decreasing (class 2), and increasing (class 3) groups. In descriptive statistics, class 1 showed the highest smoking quit rate, while class 3 showed the lowest quit rate.

In the multinomial regression, current smoking (AOR=2.28; 95% CI: 1.36–3.84) was associated with membership in the increasing alcohol dependence trajectory (class 3), while both former smoking (AOR=1.83; 95% CI: 1.24–2.70) and current smoking (AOR=2.23; 95% CI: 1.60–3.09) were associated with membership in the decreasing trajectory (class 2). The finding that current smoking predicted a worsening pattern of alcohol dependence (class 3) is consistent with prior reports that those who currently smoke are more likely to engage in problem drinking^24,25^. Moreover, the association of both former and current smoking with the decreasing alcohol dependence trajectory (class 2) suggests that those with a history of higher alcohol dependence that is now improving are more likely to have quit smoking, whereas those whose alcohol dependence is worsening over time do not show this association.

Although the weighted multinomial regression model found significant associations between class 3 and both former and current smoking, these weighted results may be less reliable. This is because subgroup size for class 3 was relatively small (e.g. only 22 participants in the age group of ≥65 years and 12 participants reporting former smoker status). Such small numbers can make weighted estimates more unstable. The unweighted model, which directly reflects the observed data, may therefore provide more stable and reliable estimates in this context.

In the fully adjusted Cox proportional hazards model, compared to class 1, those in class 2 and class 3 had a 23% and 40% lower likelihood of quitting smoking, respectively (class 2, AHR=0.77; 95% CI: 0.63–0.95; class 3, AHR=0.60; 95% CI: 0.42–0.86). These findings suggest that individuals with worsening alcohol dependence over time were the least likely to quit smoking, while those with previously high alcohol dependence that has been decreasing over time (class 2) also had lower quit rates compared to those with consistently low dependence (class 1). Importantly, the magnitude of these adjusted hazard ratios (AHRs) indicates that those in the increasing alcohol dependence trajectory (class 3) were even less likely to quit smoking than those in the decreasing trajectory (class 2).

Furthermore, the results from the stratified analysis indicate that age may modify the relationship between alcohol dependence trajectories and smoking cessation. Among adults aged 45–54 and 55–64 years, those with increasing or decreasing alcohol dependence trajectories had significantly lower quit success compared to those with consistently low trajectories, whereas this association was not significant among those aged ≥65 years. A potential explanation for this difference may be systematic differences in the reasons for wanting to quit, making quit attempts, or achieving quit success between age groups. In older adults aged >65 years, increasing numbers of comorbidities, health-related problems, or higher psychological distress have been linked to a greater likelihood of making a quit attempt or successfully quitting smoking^26^. In a qualitative study involving those with current or smoking aged >65 years, the primary reasons given for quitting smoking were health concerns^27^. In adults aged 61–75 years, the presence of a newly diagnosed chronic condition was a significant predictor of quitting smoking, but this was not the case for those aged 50–60 years^28^. In older adults, more frequent binge drinking has been associated with poorer health^29^. Therefore, drinking-related health problems may lead to smoking cessation among older adults, even though alcohol dependence generally hinders quit success.

Our LCGA results, showing that the three-class model best fits the data, are consistent with previous studies. Prior studies have also reported heterogeneity in alcohol dependence in the general population, though the number of identified subgroups has varied ^4-6^. Using NESARC III data, Linden-Carmichael et al.^30^ identified five different classes of AUD among currently drinking adults aged 18–64 years. Halonen et al.^31^ described three trajectories of risky drinking among employees approaching retirement: sustained healthy drinking, a temporary increase in risky drinking, and slowly declining drinking. Similarly, Son et al.^32^ identified four trajectories of drinking behavior using KoWePS data^32^. These differences in the number of identified classes may be due to variations in the variables entered into the LCGA models, the use of cross-sectional versus longitudinal data, or differences in the study samples, such as variations in the age ranges of participants.

Moreover, our finding that an increasing alcohol dependence trajectory is associated with reduced smoking quit rates is consistent with previous reports. In a study of elderly Koreans aged >65 years, those with AUDs were less likely to quit smoking^33^. In a study of 59018 participants who received nicotine replacement therapy through a smoking cessation program, heavy or hazardous alcohol use was associated with lower odds of successful quitting compared to low or non-use of alcohol^34^. In a study using data from National Survey on Drug Use and Health, the smoking quit rates for those with AUDs remained much lower than for those without AUDs over time^35^. Both current and past AUDs were associated with a lower likelihood of quitting smoking, while current AUDs were associated with a higher likelihood of smoking relapse^36^.

Although previous studies have primarily focused on identifying alcohol dependence subtypes using cross-sectional data and examining their association with smoking behavior, few have evaluated how subtypes based on alcohol dependence trajectories influence smoking cessation. Our analysis extends the existing literature by demonstrating not only the co-occurrence of alcohol and tobacco use but also that the trajectory of alcohol dependence affects quit outcomes. These findings suggest that aggregate analyses may obscure risks specific to subgroups if they do not account for the heterogeneity of alcohol dependence across individuals. By identifying individuals with similar longitudinal trajectories of alcohol dependence, we were able to reveal significant associations between distinct trajectories and smoking quit outcomes.

### Limitations

Limitations of this study include the potential for misclassification bias, as both alcohol use and smoking status were self-reported. Participants may have underreported socially undesirable behaviors such as heavy drinking or current smoking, leading to exposure or outcome misclassification. Because the study sample included only men aged ≥45 years, generalizability to women or younger populations is limited. Additionally, the analyses relied on data that had already been multiply imputed by the KLoSA data provider. While multiple imputation assumes data are missing at random, violations of this assumption may have biased the results. Another methodological limitation is that LCGA assumes no individual variation within classes; if within-class heterogeneity exists, model assumptions may not hold. Furthermore, important potential confounders, such as nicotine dependence, could not be included because they were not assessed in the dataset. Smoking cessation was defined based on the first reported status of former smoking, without accounting for relapse or sustained abstinence. Lastly, the three primary analyses – LCGA, multinomial logistic regression, and Cox proportional hazards modeling – were conducted on different subsamples (n=2356; n=1959; and n=1122; respectively). Although these samples were nested subsets, participants excluded due to missing data or eligibility criteria may have differed systematically, potentially introducing selection bias and influencing effect estimates. In addition, the reduction in sample size across analyses may have decreased statistical power, which could result in certain associations appearing significant in one model but not in another.

### Implications

From a policy perspective, our results emphasize the need for individualized cessation strategies based on the temporal patterns of alcohol dependence. Tobacco control policies often treat people with AUDs as a homogenous group, but our findings suggest that those who smoke cigarettes who demonstrate increasing alcohol dependence may require more intensive or comprehensive treatments. Current smoking cessation programs may be more effective if they integrate screening and interventions for concurrent alcohol dependence. Those with alcohol dependence who have repeatedly failed to quit smoking may benefit from combining behavioral and pharmacological treatments for both alcohol use disorder and smoking cessation.

### Future research

Future research should build on this analysis by further exploring the role of age as an effect modifier in the relationship between alcohol dependence trajectories and smoking cessation behaviors. This could be achieved by including younger adults to clarify how these associations differ across a broader age range. Additionally, incorporating other outcomes, such as quit intention and quit attempts, would provide a more comprehensive understanding of the association between alcohol dependence trajectories and quit behavior. Alternative study designs, like latent curve growth modeling, could be utilized to assess how alcohol dependence trajectories evolve over time. Lastly, examining interactions with variables that were not assessed in KLoSA, such as nicotine dependence, would further enhance our understanding of other factors that may potentially play an important role in the association between alcohol dependence changes and quit behavior.

## CONCLUSIONS

Few studies have examined longitudinal alcohol dependence trajectories in relation to smoking cessation. By performing LCGA, we were able to identify specific subgroups that may be particularly responsive to quitting smoking, as well as high-risk subgroups that need to be targeted by smoking cessation programs. Our study provides evidence for individualized interventions for tobacco control based on alcohol dependence trajectories. Our findings can be used to support screening for alcohol dependence in smoking cessation services for more effective tobacco control.

## Supplementary Material



## Data Availability

The data supporting this research are available from the following source: https://survey.keis.or.kr/klosa/klosadown/List.jsp The data for waves 1–7 of KLoSA (2006–2018) are available in the public domain and can be downloaded from the KLoSA website (https://survey.keis.or.kr/klosa/klosa04.jsp).
