# Supplementary material for: Alcohol dependence trajectories and smoking cessation among Korean men who smoke: A secondary data analysis from the Korean longitudinal study of aging dataset
Source: Tob Induc Dis. 2025 Jul 23;23:10.18332/tid/205795. doi: 10.18332/tid/205795 (PMC12285649; doi:10.18332/tid/205795)
Supplement: Supplementary file 1 [file TID-23-101-s1.pdf]

Supplementary File

Supplementary Table S1. Comparison of Model Fit Statistics and Class Proportions for Latent Class Growth Analysis Across Multiple Imputed Datasets (Korean Men Aged ≥45 Years, KLoSA, N=2,356)

Imputation set #	Log likelihood	BIC	AIC	Entropy	Class 1%	Class 2%	Class 3%
1	-8897.6	17888.4	17819.2	0.8	80.8	14.3	5
2	-8897.6	17888.4	17819.2	0.8	80.8	14.3	5
3	-8897.6	17888.4	17819.2	0.8	80.8	14.3	5
4	-8897.6	17888.4	17819.2	0.8	80.8	14.3	5
5	-8897.6	17888.4	17819.2	0.8	80.8	14.3	5

BIC = Bayesian Information Criterion; AIC = Akaike Information Criterion; Class1–3% = estimated class proportions.

Supplementary Table S2. Mean Posterior Probabilities by Assigned Latent Class: Classification Certainty Assessment (Korean Men Aged ≥45 Years, KLoSA, N=2,356)

Assigned Class	Mean Posterior Probability: Class 1 (Stable low)	Mean Posterior Probability: Class 2 (Decreasing)	Mean Posterior Probability: Class 3 (Increasing)
Class 1	0.95	0.037	0.014
Class 2	0.11	0.87	0.020
Class 3	0.095	0.059	0.85

The table presents the mean posterior probabilities for each assigned latent class, indicating classification certainty. Higher values along the diagonal suggest stronger classification confidence for each trajectory group.

Supplementary Table S3. Multinomial Logistic Regression Predicting Alcohol Dependence Trajectory Class Membership using Weighted Data (Korean Men Aged ≥45 Years, KLoSA, N=1,959)

Variable	Class 2 (Decreasing) (ref: Stable low)			Class 3 (Increasing) (ref: Stable low)		
	aOR	95% CI	p-value	aOR	95% CI	p-value
Age						
≥45 to <55	ref			ref		
≥55 to <65	0.88	0.72–1.07	0.20	0.96	0.83–1.10	0.55
≥65	**0.79**	**0.62–0.99**	**0.04**	0.96	0.80–1.15	0.64
Education						
Elementary school or less	ref			ref		
Middle school graduate or less than high school	0.91	0.71–1.18	0.49	0.86	0.72–1.03	0.10
High school graduate or less than college	**0.75**	**0.60–0.94**	**0.01**	**0.63**	**0.53–0.74**	**<0.001**
College graduate or above	0.94	0.72–1.22	0.63	**0.64**	**0.52–0.79**	**<0.001**
Marital status						
Married	ref			ref		
Unmarried	**1.45**	**1.08–1.95**	**0.01**	**0.68**	**0.52–0.90**	**0.01**
Employment status						
Employed	ref			ref		
Unemployed	**1.26**	**1.02–1.56**	**0.03**	1.14	0.98–1.32	0.09
Income group						
First quartile (lowest)	ref			ref		
Second quartile	1.09	0.83–1.44	0.53	1.01	0.83–1.22	0.93
Third quartile	1.19	0.91–1.54	0.21	**1.35**	**1.13–1.62**	**<0.001**
Fourth quartile (highest)	0.90	0.67–1.20	0.47	1.15	0.95–1.40	0.14
Depression						
No	ref			ref		

Yes	1.27	0.92–1.76	0.14	1.01	0.79–1.30	0.93
Self-rated health						
Good	ref			ref		
Fair	1.04	0.86–1.26	0.66	**1.63**	**1.41–1.88**	**<0.001**
Poor	**1.39**	**1.08–1.78**	**0.01**	**2.85**	**2.34–3.48**	**<0.001**
Smoking status						
Nonsmoking	ref			ref		
Former smoking	**1.78**	**1.34–2.35**	**<0.001**	**1.63**	**1.36–1.96**	**<0.001**
Current smoking	**2.04**	**1.65–2.52**	**<0.001**	**1.84**	**1.59–2.15**	**<0.001**

aOR = Adjusted Odds Ratio; CI = Confidence Interval. Income: first quartile (<300K KRW), second quartile (300–<1,200K KRW), third quartile (1,200–<3,000K KRW), fourth quartile (≥3,000K KRW). All aORs were adjusted for age, education, marital and employment status, income, depression, self-rated health, and smoking status. All confidence intervals are presented at the 95% level. Statistical significance was assessed using a two-sided p-value threshold of < 0.05.

Supplementary Table S4. Weighted Cox Proportional Hazards Model Predicting Time to Smoking Cessation by Alcohol Dependence Trajectory Class (Korean Men Aged ≥45 Years who Currently Smoke at Baseline, KLoSA, N=1,122)

Variable	Unadjusted			Adjusted		
	Hazard Ratio	95% CI	P-value	Hazard Ratio	95% CI	P-value
Class 2 (Decreasing) (ref: Stable low)	**0.69**	**0.60-0.79**	**<0.001**	**0.74**	**0.64-0.84**	**<0.001**
Class 3 (Increasing) (ref: Stable low)	**0.76**	**0.69-0.84**	**<0.001**	**0.80**	**0.72-0.88**	**<0.001**
Age 55–64 (ref: 45–54)				**1.45**	**1.32-1.59**	**<0.001**
Age ≥65 (ref: 45–54)				**1.51**	**1.33-1.72**	**<0.001**
Middle school graduate or less than high school (ref: Elementary school or less)				0.99	0.88-1.11	0.90
High school graduate or less than college (ref: Elementary school or less)				1.07	0.96-1.18	0.21
College graduate or above (ref: Elementary school or less)				**1.17**	**1.02-1.33**	**0.023**
Unmarried (ref: Married)				**0.68**	**0.57-0.81**	**<0.001**
Second quartile (ref: First quartile (lowest))				1.04	0.90-1.19	0.63
Third quartile (ref: First quartile)				1.08	0.95-1.22	0.24

Fourth quartile (ref: First quartile)				**1.46**	**1.28-1.67**	**<0.001**
Unemployed (ref: Employed)				**1.17**	**1.05-1.30**	**0.004**
Depression (ref: No depression)				1.08	0.84-1.38	0.56
Self-rated health: Fair (ref: Good)				**0.82**	**0.74-0.90**	**<0.001**
Self-rated health: Poor (ref: Good)				**0.76**	**0.67-0.87**	**<0.001**

CI = Confidence Interval. Income: first quartile (<300K KRW), second quartile (300–<1,200K KRW), third quartile (1,200–<3,000K KRW), fourth quartile (≥3,000K KRW). All aHRs were adjusted for alcohol dependence trajectory class, age, education, marital and employment status, income, depression, self-rated health. All confidence intervals are presented at the 95% level. Statistical significance was assessed using a two-sided p-value threshold of < 0.05.

Supplementary Table S5. Multinomial Logistic Regression Predicting Alcohol Dependence Trajectory Class Membership Using Complete-cases (Korean Men Aged ≥45 Years, KLoSA, N=1,382)

Variable	Class 2 (Decreasing) (ref: Stable low)			Class 3 (Increasing) (ref: Stable low)		
	aOR	95% CI	p-value	aOR	95% CI	p-value
Age						
≥45 to <55	ref			ref		
≥55 to <65	0.94	0.69-1.30	0.72	0.88	0.53-1.46	0.62
≥65	0.71	0.48-1.06	0.093	0.65	0.34-1.25	0.19
Education						
Elementary school or less	ref			ref		
Middle school graduate or less than high school	0.86	0.59-1.23	0.40	1.02	0.54-1.92	0.95
High school graduate or less than college	0.57	0.40-0.80	0.0014	1.00	0.56-1.77	1.00
College graduate or above	0.71	0.46-1.09	0.12	0.83	0.40-1.74	0.63
Marital status						
Married	ref			ref		
Unmarried	0.69	0.39-1.19	0.18	1.74	0.90-3.38	0.099
Employment status						
Employed	ref			ref		
Unemployed	0.96	0.70-1.31	0.79	1.16	0.70-1.94	0.56
Income group						
First quartile (lowest)	ref			ref		
Second quartile	**1.49**	**1.01-2.21**	**0.044**	0.8	0.42-1.52	0.50
Third quartile	**1.84**	**1.25-2.69**	**<0.01**	1.25	0.70-2.24	0.45
Fourth quartile (highest)	1.11	0.72-1.70	0.65	0.89	0.46-1.70	0.71
Depression						
No	ref			ref		

Yes	**1.86**	**1.14-3.02**	**0.012**	1.99	0.94-4.24	0.074
Self-rated health						
Good	ref			ref		
Fair	**1.39**	**1.04-1.86**	**0.027**	0.99	0.60-1.61	0.96
Poor	**1.97**	**1.34-2.88**	**<0.001**	1.47	0.78-2.78	0.24
Smoking status						
Nonsmoking	ref			ref		
Former smoking	**1.83**	**1.24-2.71**	**<0.01**	0.95	0.45-1.97	0.88
Current smoking	**2.25**	**1.62-3.12**	**<0.001**	**2.27**	**1.35-3.83**	**<0.01**

aOR = Adjusted Odds Ratio; CI = Confidence Interval. Income: first quartile (<300K KRW), second quartile (300–<1,200K KRW), third quartile (1,200–<3,000K KRW), fourth quartile (≥3,000K KRW). All aORs were adjusted for age, education, marital and employment status, income, depression, self-rated health, and smoking status. All confidence intervals are presented at the 95% level. Statistical significance was assessed using a two-sided p-value threshold of < 0.05.

Supplementary Table S6. Cox Proportional Hazards Model Predicting Time to Smoking Cessation Using Complete-cases (Korean Men Aged ≥45 Years Who Currently Smoke at Baseline, KLoSA, N=938)

Variable	Unadjusted			Adjusted		
	Hazard Ratio	95% CI	P-value	Hazard Ratio	95% CI	P-value
Class 2 (Decreasing) (ref: Stable low)	0.82	0.65-1.04	0.10	0.87	0.68-1.10	0.25
Class 3 (Increasing) (ref: Stable low)	**0.66**	**0.44-0.99**	**0.044**	0.70	0.47-1.06	0.09
Age 55–64 (ref: 45–54)				**1.35**	**1.09-1.67**	**0.01**
Age ≥65 (ref: 45–54)				**1.62**	**1.21-2.16**	**<0.001**
Middle school graduate or less than high school (ref: Elementary school or less)				1.08	0.81-1.43	0.59
High school graduate or less than college (ref: Elementary school or less)				1.22	0.95-1.56	0.13
College graduate or above (ref: Elementary school or less)				1.33	0.98-1.81	0.07
Unmarried (ref: Married)				0.69	0.47-1.02	0.07
Second quartile (ref: First quartile (lowest))				0.89	0.68-1.17	0.41
Third quartile (ref: First quartile)				0.87	0.66-1.14	0.30

Fourth quartile (ref: First quartile)				1.2	0.91-1.57	0.20
Unemployed (ref: Employed)				1.19	0.95-1.49	0.13
Depression (ref: No depression)				1.17	0.80-1.70	0.42
Self-rated health: Fair (ref: Good)				0.96	0.79-1.17	0.69
Self-rated health: Poor (ref: Good)				0.84	0.62-1.14	0.27

CI = Confidence Interval. Income: first quartile (<300K KRW), second quartile (300–<1,200K KRW), third quartile (1,200–<3,000K KRW), fourth quartile (≥3,000K KRW). All aHRs were adjusted for alcohol dependence trajectory class, age, education, marital and employment status, income, depression, self-rated health. All confidence intervals are presented at the 95% level. Statistical significance was assessed using a two-sided p-value threshold of < 0.05.

Supplementary Table S7. Stratified Cox Proportional Hazards Models Predicting Smoking Cessation by Age Group (Korean Men Aged ≥45 Years Who Currently Smoke at Baseline, KLoSA, N=1,122)

	45-54 years		55-64 years		≥65 years	
Alcohol dependence trajectory class	aHR (95% CI)	p-value	aHR (95% CI)	p-value	aHR (95% CI)	p-value
Class 1	ref		ref		ref	
Class 2	**0.69 (0.50-0.96)**	**0.027**	**0.67 (0.47-0.96)**	**0.031**	1.17 (0.77-1.80)	0.46
Class 3	**0.47 (0.28-0.79)**	**0.004**	0.64 (0.31-1.32)	0.23	1.10 (0.54-2.23)	0.80

CI = Confidence Interval. All aHRs were adjusted for alcohol dependence trajectory class, education, marital and employment status, income, depression, self-rated health. All confidence intervals are presented at the 95% level. Statistical significance was assessed using a two-sided p-value threshold of < 0.05

© 2025 Han M. et al.
